# Assessment of the safety and probiotic characteristics of *Lactobacillus salivarius* CGMCC20700 based on whole-genome sequencing and phenotypic analysis

**DOI:** 10.3389/fmicb.2023.1120263

**Published:** 2023-03-16

**Authors:** Yu-Hang Jiang, Rui-Si Yang, Yi-Cen Lin, Wei-Gang Xin, Huan-Yu Zhou, Feng Wang, Qi-Lin Zhang, Lian-Bing Lin

**Affiliations:** ^1^Faculty of Life Science and Technology, Kunming University of Science and Technology, Kunming, Yunnan, China; ^2^College of Food Science, Southwest University, Chongqing, China; ^3^Engineering Research Center for Replacement Technology of Feed Antibiotics of Yunnan College, Kunming, Yunnan, China

**Keywords:** *Lactobacillus salivarius*, whole-genome sequence, phenotypic analysis, safety, probiotic characteristics

## Abstract

Lactic acid bacteria are generally regarded as alternatives to antibiotics in livestock and poultry farming, especially *Lactobacillus* strains, which are safe and have probiotic potential. Although *Lactobacillus salivarius* has long been proposed to be a probiotic, the understanding of the roles of this species is still in its infancy. Here, a strain of *L. salivarius* CGMCC20700 isolated from the intestinal mucosa of Yunnan black-bone chicken broilers was investigated in the context of its safety and probiotic characteristics by whole-genome sequencing in parallel with phenotypic analysis. Whole-genome sequencing results showed that *L. salivarius* CGMCC20700 has a single scaffold of 1,737,577 bp with an average guanine-to-cytosine (GC) ratio of 33.51% and 1,757 protein-coding genes. The annotation of Clusters of Orthologous Groups (COG) classified the predicted proteins from the assembled genome as possessing cellular, metabolic, and information-related functions. Sequences related to risk assessment, such as antibiotic resistance and virulence genes, were identified, and the strain was further confirmed as safe according to the results of antibiotic resistance, hemolytic, and acute oral toxicology tests. Two gene clusters of antibacterial compounds and broad-spectrum antimicrobial activity were identified using genome mining tools and antibacterial spectrum tests. Stress resistance genes, active stressor removal genes, and adhesion related genes that were identified and examined with various phenotypic assays (such as stress tolerance tests in acids and bile salts and auto aggregation and hydrophobicity assays). The strain showed a high survival rate in the presence of bile salts and under acidic conditions and exhibited significant auto aggregation capacity and hydrophobicity. Overall, *L. salivarius* CGMCC20700 demonstrated excellent safety and probiotic potential at both the genomic and physiological levels and can be considered an appropriate candidate probiotic for livestock and poultry farming.

## Introduction

For decades, antibiotics have been widely used in livestock and poultry farming-related fields and are commonly used to prevent or treat bacterial infections and as antimicrobial growth promoters (Al-Khalaifa et al., 2019). The United Nations Food and Agriculture Organization (FAO) reported that the total amount of antibiotics used in farming processes such as poultry, animal husbandry, and aquaculture is alarming (Pierce et al., 2020). The global usage of antibiotics in food animals reached 93,309 ton in 2017 and was projected to increase by 11.5% to 104,079 tons by 2030 (Tiseo et al., 2020). However, the widespread use of antibiotics has created problems, including animal gut microbiome disorders, the development of antibiotic resistance, and environmental issues (Al-Khalaifa et al., 2019; Ramirez et al., 2020; Tiseo et al., 2020). For instance, previous studies have shown that vancomycin treatment in mice can promote the proliferation of pathogenic gram-negative bacteria, leading to an imbalance in the gut microbiome (Yamaguchi et al., 2020). In a study of *Enterococcus* in fecal samples from broilers fed antibiotics, VanA transposons were found to be transported from animals to humans (Van et al., 2002). Many countries have now promulgated laws to ban the nontherapeutic use of antibiotics in poultry and livestock farming processes, including the European Union, United States of America, and China (FDA, 2009; Ricke et al., 2020). Therefore, there is an urgent need to find effective alternatives to antibiotics for application in the livestock and poultry industries.

Probiotics are defined by the WHO/FAO as “live microorganisms,” which can provide health benefits to the host when given in sufficient amounts (García-Hernández et al., 2016; Kai et al., 2020). For example, dietary supplementation with *Lactobacillus fermentum* in a mouse model of colitis was proven to initiate signaling pathways involved in epithelial barrier protection (Paveljšek et al., 2018). Similarly, supplementation with *Lactobacillus rhamnosus* GG and *Lactobacillus casei* IMAU60214 triggered innate immune responses and improved the phagocytic and bactericidal activities of human macrophages (Rocha-Ramírez et al., 2017). Moreover, many probiotics have been shown to have positive effects in livestock and poultry farming processes (Cerezuela et al., 2012; Hiremath and Pragasam, 2022). *Bacillus subtilis* feeding for 2 weeks enhanced the serum IgM level and leukocyte phagocytosis activity in gilthead seabream (Cerezuela et al., 2012). *Lacticaseibacillus paracasei* NSMJ56 feeding for 10 days increased the abundance of CD4+ T cells in the small intestinal lamina propria and gut microbial diversity in early-age broiler chickens (Joo et al., 2022). Therefore, this evidence indicates that probiotics can serve as functional microbiological resources for use as alternatives to antibiotics.

*Lactobacillus salivarius*, an important member of lactic acid bacteria (LAB), is widely distributed in traditional fermented food and animal gastrointestinal tracts, particularly in the avian intestine (Chiu et al., 2017; Li H. W. et al., 2021). Previous studies have shown that *L. salivarius* strains, as potential probiotic strains, possess inhibitory activity against intestinal pathogens and regulate the balance of the intestinal microbiome due to the production of many selectively stimulating metabolites, as well as antimicrobial compounds, antioxidants, and organic acids (Chen et al., 2022; Xu et al., 2022). For instance, in broilers, *L. salivarius* Erya conferred resistance to *Salmonella pullorum* infection and alleviated the negative effects of aflatoxin B1, while adding *L. salivarius* Erya also improved growth performance, liver function, and meat quality (Chen et al., 2022); in laying hens, *L. salivarius* CML352 was considered a suitable probiotic with positive effects on intestinal health and performance (Xu et al., 2022). These scattered examples indicate that *L. salivarius* is possibly a probiotic species. However, in recent years, a growing body of research has revealed that the functions of probiotics are highly strain specific and that their biological effects should be individually evaluated (Tanizawa et al., 2015; Li et al., 2020). Particularly, for newly isolated probiotics, it is necessary to analyze and evaluate the related characteristics of their probiotic function at the gene level to explore more potential biological functions and information (Tanizawa et al., 2015; Saroj and Gupta, 2020).

Although many whole genome sequences of LAB probiotics have been reported, their whole genome participation and *in vivo* probiotic effects are still poorly understood (Goel et al., 2020; Qureshi et al., 2020; Saroj and Gupta, 2020). In our previous study, a strain of *L. salivarius* CGMCC20700 with significant antibacterial ability was isolated from the intestinal mucosa of Yunnan black-bone chicken broilers and confirmed to produce active antimicrobial substances as a novel bacteriocin (Li H. W. et al., 2021). However, the safety level, probiotic capabilities, and practical potential for this strain to be used as an alternative to antibiotics remain unknown. Therefore, the aim of this study was to evaluate the safety and potential probiotic characteristics of *L. salivarius* CGMCC20700 using a series of *in vitro* tests. Additionally, the whole genome sequence was analyzed to provide a deeper understanding and insight into the full breadth of its biological capabilities for an assessment of safety and probiotic-associated capacity.

## Materials and methods

### Bacterial strains and growth conditions

*Lactobacillus salivarius* CGMCC20700 was isolated from the intestinal mucosa of Yunnan black-bone chickens (*Gallus gallus*) and is deposited at the China General Microbiological Culture Collection Center (CGMCC). The strain was cultured in de Man Rogosa Sharpe (MRS) medium (Solarbio, Beijing, China) at 37°C for 24 h in anaerobic jars for routine use, as previously reported (Li H. W. et al., 2021).

### Identification of *Lactobacillus salivarius* CGMCC20700

The morphology and phylogenetics of *L. salivarius* CGMCC20700 were assessed as previously reported (Fu et al., 2022; Jiang et al., 2022a). Briefly, *L. salivarius* CGMCC20700 was cultured on MRS solid medium plates at 37°C for 24 h, the colonies on the plates were observed, Gram staining was conducted, and the bacterial cell morphology was observed by a scanning electron microscope (S-3000 N, Hitachi) (Jiang et al., 2022a). Finally, the genotypic identification of *L. salivarius* CGMCC20700 was conducted by comparison of its 16S rRNA sequence analysis with the sequences deposited in the National Center Biotechnology Information (NCBI) database using the Basic Local Alignment Search Tool (BLAST). The phylogenetic tree was reconstructed using MEGA6 software with the neighbor-joining method (Tamura et al., 2011).

### Whole genome sequencing, assembly, and annotation

Whole-genome sequencing, assembly and annotation of *L. salivarius* CGMCC20700 were conducted by Beijing Genomics Institute (Shenzhen, China). Briefly, *L. salivarius* CGMCC20700 was cultured to exponential phase and collected by centrifugation at 8000 × *g* for 5 min, and the total DNA of bacterial cells was extracted using a DNA Purification kit (Solarbio, Beijing, China). Subsequently, whole-genome sequencing was carried out using a combination of the second-generation BGISEQ platform and the third-generation PacBio platform sequencing technology (Huada Gene Co., Ltd., Shenzhen, China) (Dai et al., 2021; Gao et al., 2021). The assembly of the completed sequence was performed by using GATK v. v1.6–13 and SMRT Analysis v. v2.2.0 software to assemble the main complete and continuous contigs. After single-base correction, loop judgment and other analyzes based on the obtained contigs, we generated credible complete map sequences. The genome annotation of *L. salivarius* CGMCC20700 was performed using the Prokaryotic Genome Annotation Pipeline (PGAP) algorithm of the National Center for Biotechnological Information (NCBI) (Kai et al., 2020; Vyacheslav et al., 2020). Then, GeneMark software (V4.17)^1^ was adopted for the prediction of protein-coding RNA in the whole genome. The prediction of sRNA, rRNA, and tRNA was determined using the cmsearch program V1.1rc4, RNAmmer 1.2 and tRNAscan-SE V1.3.1. The CRISPR regions were identified using CRISPR digger V1.0, and plasmid information was obtained using an online tool with Plasmid Finder. The Cluster of Orthologous Groups of Proteins (COG) database was used for general function annotation. The genome sequences of *L. salivarius* CGMCC20700 have been submitted to GenBank under accession number CP101685.

### Safety assessment of *Lactobacillus salivarius* CGMCC20700

#### Identification of safety-related genes

Safety-related genes were identified to evaluate the potential safety at the genomic level of *L. salivarius* CGMCC20700, as previously reported (Fu et al., 2022; Wu et al., 2022). Putative virulence genes of *L. salivarius* CGMCC20700 were analyzed by comparison with the VFDB. Antibiotic resistance genes of *L. salivarius* CGMCC20700 were identified using the ARDB.

#### Antibiotic resistance analysis

Antibiotic resistance was evaluated by adopting the method from a previous study (Zheng et al., 2021). Briefly, susceptibility to the following 13 antibiotics was assessed using filter paper disks infused with the following: 30 μg each of ceftazidime, cefuroxime, cefazolin, vancomycin, and tetracycline; 10 μg each of penicillin, streptomycin, gentamicin, and amoxicillin; 100 μg of ampicillin; and 15 μg of erythromycin (Shanghai Yibaiju Economic and Trade Co., Ltd., Shanghai, China). A volume of 100 μl of *L. salivarius* CGMCC20700 cultures (10^7^ CFU/mL) was spread on MRS solid medium plates, and the antibiotic-infused paper disks were adhered to the surface of MRS medium and cultured at 37°C for 24 h. The inhibition zones (mm) were measured using a Vernier caliper. According to the guidelines of the Institute of Clinical and Laboratory Standards Institute (CLSI), the drug resistance susceptibility was determined as follows: *S* = sensitive (zone diameter ≥ 17 mm); I = intermediate (zone diameter 12 to 17 mm); *R* = resistant (zone diameter ≤ 1.2 cm).

#### Hemolytic activity analysis

The hemolytic activity assays were performed by adopting the method from Zheng et al. (2021). Briefly, *L. salivarius* CGMCC20700 and *Escherichia coli* CMCC(B)44102 cultures were crossed, streaked on Columbia blood agar containing fresh sheep blood (Shanghai Yibaiju Economic and Trade Co., Ltd., Shanghai, China) and incubated at 37°C for 24 h. Hemolytic activity was determined according to the following rules: if the colony (strain) showed a grass-green ring on the plate, the strain was identified as α-hemolytic; if the colony showed a completely clear hemolytic ring on the plate, the strain was identified as β-hemolytic; and if no changes were observed, the colony was identified as nonhemolytic.

#### Broiler acute toxicity assay

An acute toxicity assay was conducted to assess the safety of *L. salivarius* CGMCC20700 in broilers. Briefly, *L. salivarius* CGMCC20700 cultures (10^7^ CFU/mL) were harvested by centrifugation (8,000 × *g*, 5 min), washed several times with sterile water, mixed well with freeze-dried protection solutions (10% trehalose and 10% skim milk) and then freeze-dried to the desired concentration (1× 10^10^ CFU/g). Twenty healthy broilers (half male, 3 days old) were provided by Kunming Yuankang Food Agriculture and Animal Husbandry Co., Ltd. The freeze-dried *L. salivarius* CGMCC20700 powders were prepared with sterile water to a concentration of 2 g/mL, and a 20 mL/kg body weight dose was gavaged two times a day at 4-h intervals after a 6-h fast. The diet composition and housing conditions for broiler feeding were followed as presented in our previous study (Jiang et al., 2022b). The experiment lasted for 14 days, and internal tissues and organs were immediately observed and evaluated by the naked eye after exposure.

### Assessment of probiotic properties

#### Analysis of antimicrobial compounds in the genome

Antimicrobial genes were identified to confirm the antimicrobial ability of *L. salivarius* CGMCC20700, as previously reported (Goel et al., 2020; Zheng et al., 2021). The presence of gene clusters of nonribosomally synthesized secondary metabolites (NRPS) was evaluated using AntiSMASH 5.^2^ The potential bacteriocin synthesis gene clusters were identified using the BAGEL4 webserver.^3^

#### Assessment of antimicrobial spectrum

The antimicrobial activity of *L. salivarius* CGMCC20700 against six common pathogen indicator strains was determined as previously reported (Jiang et al., 2022a). Briefly, all indicator strains (as shown in Table 1) were precultured in Luria-Bertani (LB) liquid medium (Solarbio, Beijing, China) at 37°C for 12 h. Later, the antimicrobial activity of the *L. salivarius* CGMCC20700 cell-free supernatant (200 μL) against each indicator strain (10^7^ CFU/mL) was determined by the Oxford cup double-plate method. The inhibition zone was measured using a Vernier caliper. MRS broth medium was used as a control.

**Table 1:** General genome features of the *Lactobacillus salivarius* CGMCC20700 genome.

Attribute	Value
Genome size (bp)	1,929,539
GC content (%)	33.51
Plasmid	2
Total RNA	105
Number of rRNAs	22
Number of tRNAs	78
Number of ncRNAs	5
Number of protein-coding genes	1,757
Number of prophage region	5

#### Identification of probiotic-related genes in the genome

Probiotic genes were identified to evaluate the potential probiotic functions of *L. salivarius* CGMCC20700, as previously reported (Goel et al., 2020; Zheng et al., 2021). Briefly, different probiotic genes were obtained using BLASTP in the NCBI database and compared with known probiotic genes. Subsequently, the genes of *L. salivarius* CGMCC20700 were classified into functions related to stress resistance, DNA and protein protection and repair, active removal of stressors, antipathogenic effects, immunomodulation, and adhesion ability.

#### Bile salt and acid tolerance assay

The bile salt and acid tolerance assay were performed by adopting the method described by Li X. Y. et al. (2021). Briefly, for bile salt tolerance tests, different concentrations (0.3, 0.6, and 0.9% (w/v)) of bile salts (Sangon Biotech, Shanghai, China) were added to MRS broth medium; for acid resistance tests, MRS broth medium was adjusted to different pH values (pH 2.0, 3.0 and 4.0) using 2 mol/mL HCl. Subsequently, *L. salivarius* CGMCC20700 cultures (10^7^ CFU/mL) were added to MRS broth medium and cultured at 37°C for 4 h and 5 h, respectively. After incubation, 100 μL of each bacterial suspension was separately coated on MRS solid plates by the serial dilution method and inverted incubation at 37°C for 24 h. The viable counts of 30 ~ 300 colonies were counted, and bile salt tolerance was determined by calculating the ratio (%) of viable cells compared to the control without bile salt survival rates (%).

#### Auto aggregation capacity assay

The auto aggregate capability was determined according to the method described by Qureshi et al. (2020). Briefly, *L. salivarius* CGMCC20700 was added to MRS broth medium, and after culturing at 37°C for 12 h, the cells were collected by centrifugation at 8000 × *g* for 10 min, washed twice with PBS (pH 7.0), and resuspended to OD_600_ = 0.6. The initial absorbance value (Ab0) was measured. Then, the absorbance of 1 mL of bacterial suspension from each Eppendorf (EP) tube was measured as the OD_600_ value (Abt) of the supernatant after allowing it to stand at 37°C for 2–3 h. The percentage of auto aggregation was as follows: Auto aggregation(%)=(Ab0−Abt)/Ab0×100 .

#### Cell surface hydrophobicity

The hydrophobicity of bacteria was determined according to the method described by Qureshi et al. (2020), with slight modifications. Briefly, *L. salivarius* CGMCC20700 was cultured in MRS broth at 37°C for 12 h and then centrifuged at 8000 × *g* for 10 min to collect the cells. The pellet was washed twice with PBS buffer, and the cells were resuspended in PBS to a cell OD_600_ = 0.7. The initial absorbance (Abi) was recorded. Afterward, the bacterial suspension was mixed with xylene (3:1) and incubated at 37°C for 10 min. The mixture was left standing at 37°C for 1 h, the aqueous phase was separated, and its absorbance (Abf) was measured at 600 nm. Surface hydrophobicity was calculated according to the following formula: Hydrophobicity(%)=100×(Abi−Abf)/Abi.

### Statistical analysis

All experiments were conducted in triplicate, and each sample was evaluated in triplicate. The results are presented as the mean ± standard deviation (SD). Statistical significance was determined by one-way analysis of variance (ANOVA) in SPSS 22.0 statistical software (IBM Software Inc., NY, United States). *p* values <0.05 were considered indicative of a significant difference.

## Results

### Identification of *Lactobacillus salivariu*s CGMCC20700

**Figure 1:**
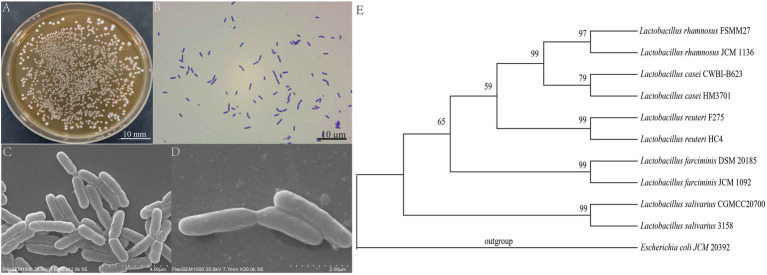
Morphological identification and phylogenetic tree of *Lactobacillus salivarius* CGMCC20700. **(A)** Colony observation, **(B)** Observation of Gram staining, **(C**,**D)** SEM observation, and **(E)** phylogenetic tree. All sequences originated from *Lactobacillus* strains, and other *Lactobacillus* species were used as outgroups. The numbers at the nodes indicate the bootstrap values of neighbor joining analyzes with 1,000 replicates.

The results of morphological identification showed that the *L. salivarius* CGMCC20700 strain had colonies with a round, medium-sized, raised, whitish, moist, entire edge (Figure 1A). The *L. salivarius* CGMCC20700 strain is gram-positive (Figure 1B) and arranged in short rods without spores or flagella under SEM observation (Figures 1C,D). Based on 16S rRNA analysis, the *L. salivarius* CGMCC20700 strain showed ≥99% similarity with the *L. salivarius* 3,158 strain. The phylogenetic tree was constructed using the neighbor-joining method in MEGA 6 software (Figure 1E).

### Genome properties of *Lactobacillus salivarius* CGMCC20700

**Figure 2:**
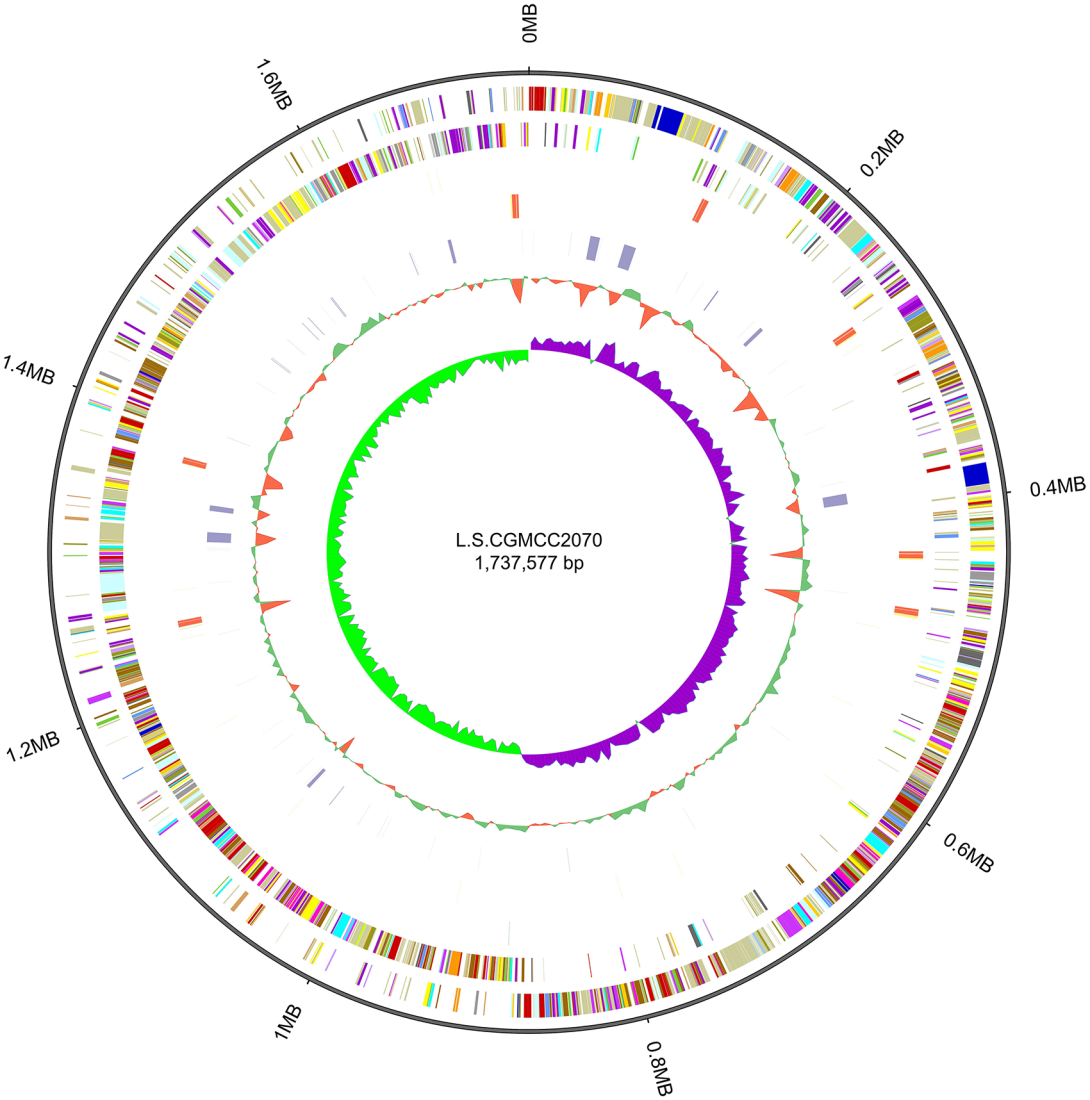
Circular genome map of *L. salivarius* CGMCC20700.

**Figure 3:**
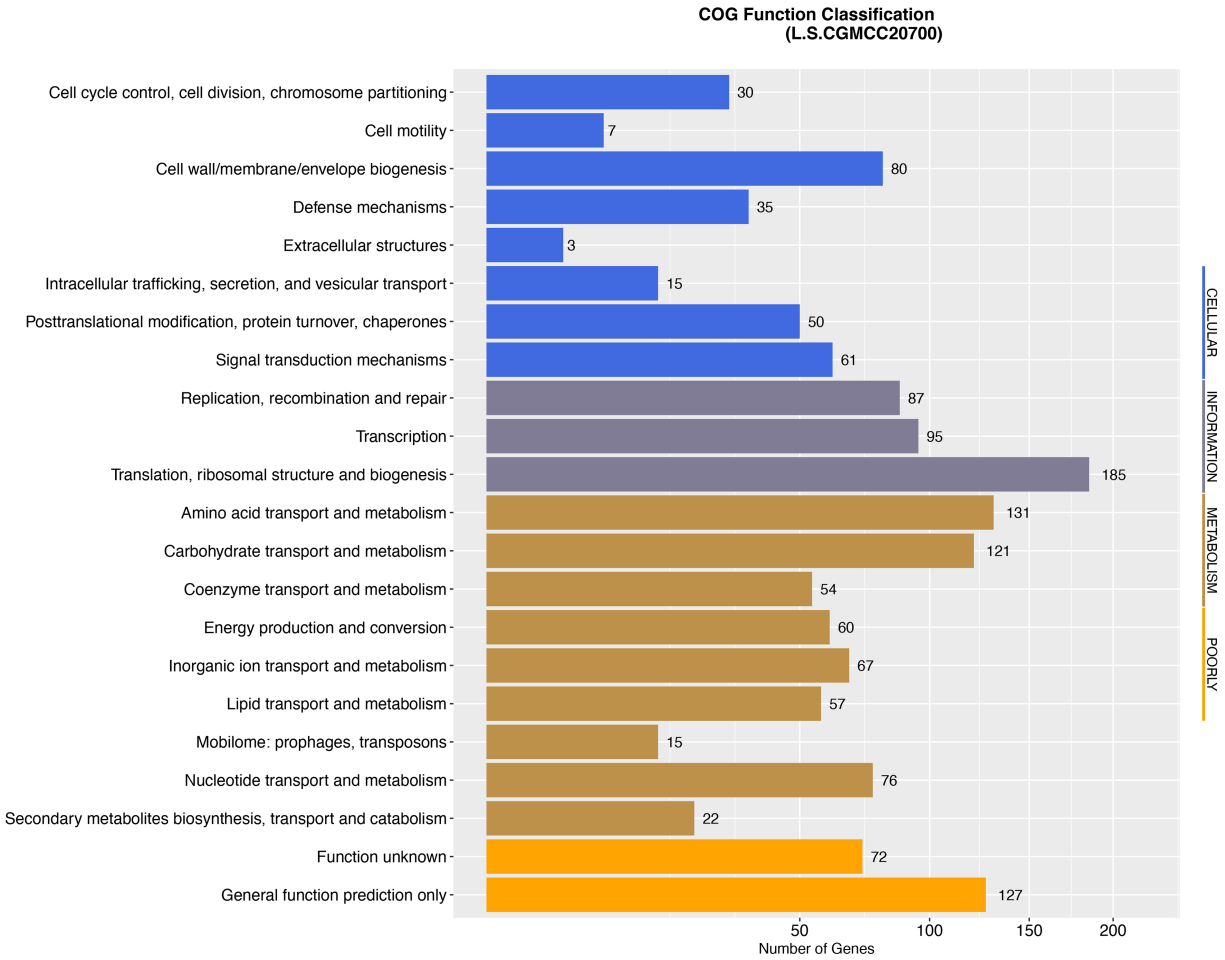
Distribution of genes across COG functional categories in the genome of *L. salivarius* CGMCC20700.

Whole-genome sequencing of *L. salivarius* CGMCC20700 showed that its genome size was 1.92 Mb with a single, circular chromosome with a GC content of 33.51% and two circular plasmids named plasmid1 (169,139 bp) and plasmid2 (22,823 bp) (Figure 2), which matched the results reported by Chiu et al. (2017). A total of 1,757 protein-coding sequences, 78 tRNA genes, 22 rRNA genes and 5 sRNA genes were identified, as shown in Table 1. Based on the COG database, 1,450 protein-coding genes were assigned to families comprising 22 functional categories into four types, including cellular, metabolism, information, and assembled genome. COG classification showed that *L. salivarius* CGMCC20700 was involved in the following aspects: (1) translation/ribosomal structure and biogenesis, (2) amino acid transport and metabolism, (3) carbohydrate transport and metabolism, (4) energy production and conversion, (5) coenzyme transport and metabolism, and (6) secondary metabolite biosynthesis, transport, and catabolism (Figure 3).

### Safety analysis of *Lactobacillus salivarius* CGMCC20700

#### Identification of antibiotic resistance and toxicological factors

**Figure 4:**
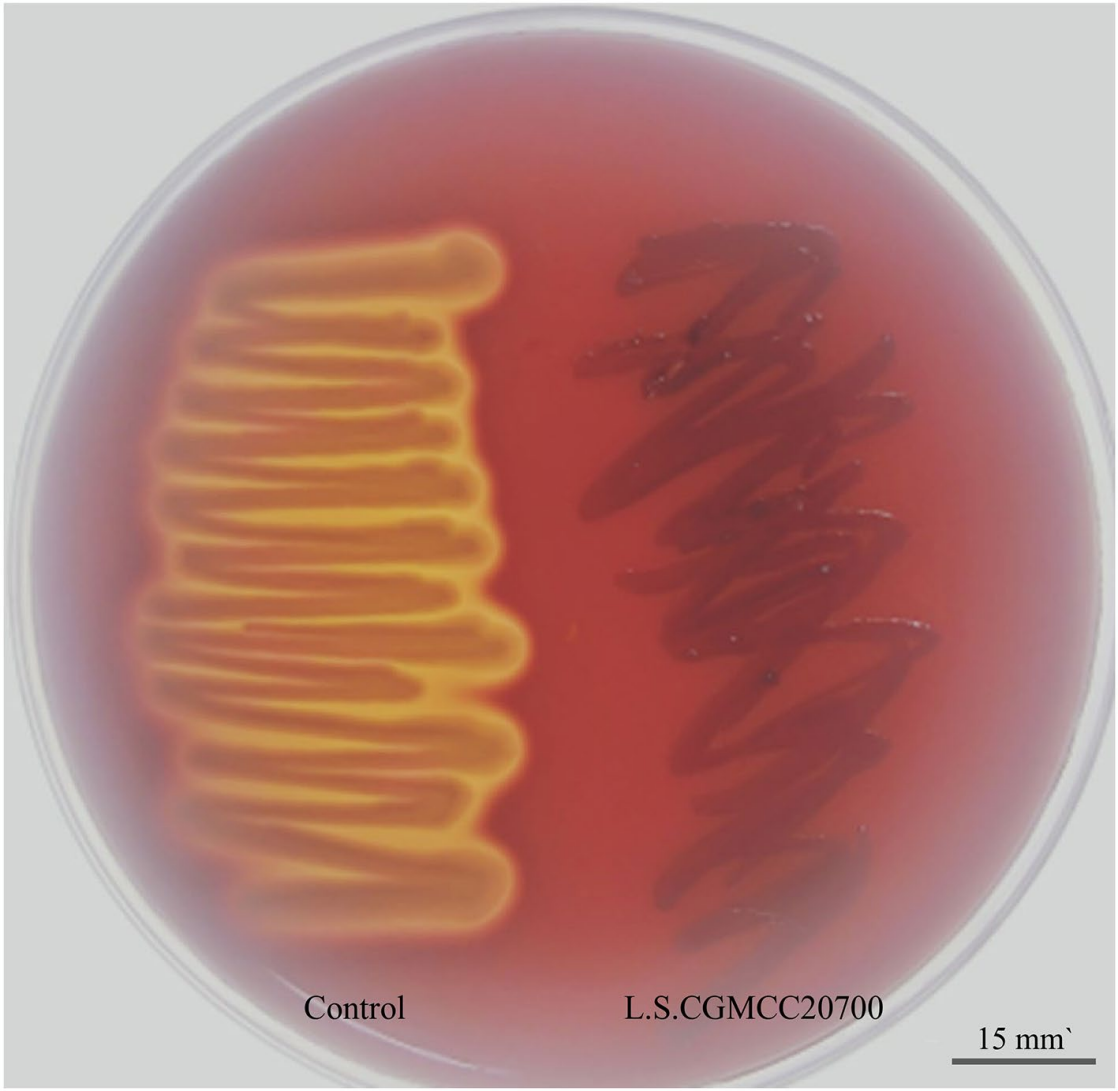
Hemolysis ability of *L. salivarius* CGMCC20700. The positive control *Escherichia coli* CMCC(B)44102 produced an obvious zone of β-hemolysis (left); CGMCC20700 showed γ-hemolysis (right).

The genes related to antibiotic resistance and toxin production in the *L. salivarius* CGMCC20700 genome were identified according to the VFDB and ARDB databases, respectively. Based on the ARDB, 10 genes associated with antibiotic resistance were identified, and only three resistance genes with more than 90% similarity were covered, including tetracycline (*tetm*, *tetl*) and macrolide (*ermc*) resistance-related genes (Supplementary Table S1). Meanwhile, a total of 83 putative virulence factor genes were identified based on the VFDB database. The similarity of most putative virulence factor genes with VFDB was less than 80% (Supplementary Table S2). Furthermore, *L. salivarius* CGMCC20700 was sensitive to the antibiotics tested, as shown in Table 2, and was found to be sensitive only to vancomycin and erythromycin. The positive control bacteria (*E. coli* CMCC(B)44102) showed significant inhibition zones, which were identified as β hemolysis, while the *L. salivarius* CGMCC20700 strain did not show any hemolytic activity (Figure 4).

**Table 2:** Antibiotic resistance of *L. salivarius* CGMCC20700.

Antibiotic	Interpretation
Ceftazidime	S
Cefuroxime	S
Cefazolin	S
Vancomycin	S
Tetracycline	R
Penicillin	S
Streptomycin	S
Gentamicin	I
Amoxicillin	S
Ampicillin	S
Erythromycin	R

“*S*”, Susceptible; “*I*”, Intermediate; “*R*”, Resistant.

#### Broiler acute toxicity test

The broilers were fed 20 g/kg body weight *L. salivarius* CGMCC20700 solution every day. The weight of broilers gradually increased during 14 days of observation, and no poisoning or mortality was found, as shown in Table 3. After the experiment, the broilers were dissected, and no internal tissue and organ lesions were observed by the naked eye.

**Table 3:** Results of the acute oral toxicity test.

Animal sex	Dose (g/kg bw)	Text Animals (*n*)	Weight (X ± SD) (g)			Dead Animals (*n*)	Death Rate (%)
			Day 0	Day 7	Day 14		
Male	20	10	46.73 ± 0.79	114.53 ± 3.59	237.63 ± 5.87	0	0
Female	20	10	47.77 ± 0.76	120.07 ± 3.95	259.70 ± 4.74	0	0

### Assessment of probiotic properties

#### Antimicrobial compound genes

**Figure 5:**
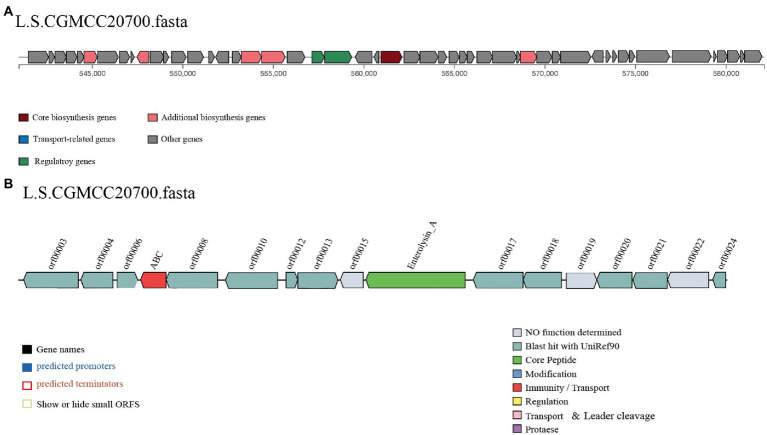
Predicted biosynthetic gene clusters encoding antibacterial compounds in the *L. salivarius* CGMCC20700 genome. The gene clusters encoding T3PKS **(A)** and enteroccin A **(B)** are represented by arrows with different colors corresponding to the operons of different functions.

The AntiSMASH 5.0 and BAGEL 4.0 databases were used to identify putative genes in the *L. salivarius* CGMCC20700 genome involved in antimicrobial compound production. In the two databases, two genes associated with T3PKS and enterococcin A were identified by antiSMASH and BAGEL4, respectively (Figure 5). Meanwhile, the cell-free supernatant of *L. salivarius* CGMCC20700 showed significant antibacterial activity against selected common pathogenic bacteria, both Gram-positive and Gram-negative, compared with the control (*p* < 0.01), particularly against *Staphylococcus aureus* ATCC2592, *Staphylococcus sciuri* ATCC 29059 and *Salmonella enteritidis* CMCC (B) 50,335, with an inhibitory zone reaching up to 24 mm (Table 4).

**Table 4:** Antibacterial spectrum of *L. salivarius* CGMCC20700.

Selected bacteria	Medium and temperature (°C)	Inhibition zone (mm)
Control		8.03 ± 0.01
Gram-positive bacteria		
*Staphylococcus aureus* ATCC2592	LB, 37	26.65 ± 0.59^**^
*Streptococcus agalactiae* CMCC(B)32,116	LB, 37	25.47 ± 0.22^**^
*Staphylococcus sciuri* ATCC 29059	LB, 37	24.47 ± 0.25^**^
*Gram-negative bacteria*		
*Shigella flexneri* CICC 21678	LB, 37	25.27 ± 0.19^**^
*E. coli* ATCC 3521	LB, 37	21.35 ± 0.09^**^
*Salmonella enteritidis* CMCC (B) 50,335	LB, 37	22.39 ± 0.23^**^

^*^p < 0.05; ^**^p < 0.01.

#### Identification of probiotic genes

The genes related to probiotic properties were identified by annotation in the whole genome of *L. salivarius* CGMCC20700, as shown in Table 5. Of these, genes responsible for stress resistance included *dltA*, *dltD* and *dnaK*; genes responsible for DNA and protein protection and repair included *folC*, *aclp L* and *msr B*; genes responsible for the active removal of stressors included *rfbB* and *bsh*; genes responsible for immunomodulation included *dlt B* and *dlt D*; and genes responsible for anti-pathogenic effects included *Lux S*. Genes responsible for adhesion ability included *Mucin22* and *fbp*. In addition to the adhesive ability-related gene *Mucin22*, the similarity of all other genes related to probiotic properties was over 98%.

**Table 5:** Probiotic characteristics of *L. salivarius* CGMCC20700-related annotated genes.

Gene	Response	Locus tag	Identity (%)
Stress resistance genes			
*dltA* (*L. plantarum*)	Acid and defensin resistance	L.S.GL000791	99.80
*dltD* (*L. rhamnosus*)	Acid and defensin resistance	L.S.GL000788	99.29
*dnaK* (*L. salivarius*)	Heat shock tolerance	L.S.GL000512	99.84
DNA and protein protection and repair			
*folC* (*L. salivarius*)	Nucleic acid biosynthesis required for host fetal nervous system growth	L.S.GL000937	98.85
*clp L* (*L. reuteri*)	Acid and bile tolerance	L.S.GL000082	98.86
*clp C* (*L. plantarum*)	Persistence capacity *in vivo*	L.S.GL000212	99.88
*msr B* (*L. salivarius*)	Persistence capacity *in vivo*	L.S.GL000034	100%
Active removal of stressors			
*rfbB* (*L. salivarius*)	low pH tolerance	L.S.GL001405	99.71
*bsh* (*L. salivarius*)	Bile salt resistance	L.S.GL001662	99.07
Anti-pathogenic effect			
Lux S (*L. salivarius*)	Autoinduction ability	L.S.GL001051	100
Immunomodulation			
*dlt B* (*L. salivarius*)	Anti-inflammatory potential *in vitro* in PBMCs and *in vivo* in a murine model of colitis	L.S.GL000790	99.75
*dlt D* (*L. salivarius*)	Resistance to human β-defensin-2	L.S.GL000788	99.29
Adhesion ability			
*Mucin22*	Adhesion ability	L.S.GL000167	92.40
*fbp*	Adhesion ability	L.S.GL000773	99.64

#### Bile salt and acid tolerance

The treatment results of CGMCC20700 at different concentrations of acid-resistant and bile salts are shown in Table 6. When treated with bile salt concentrations of 0.3, 0.6 and 0.9% for 4 h, the survival rate of *L. salivarius* CGMCC20700 significantly decreased to 62.64, 33.76, and 26.01%, respectively, compared with the control (*p* < 0.05). At pH values of 2, 3 and 4 for 5 h, the survival rate of *L. salivarius* CGMCC20700 decreased to 57.91% (*p* < 0.05), 83.91% (*p* < 0.05) and 92.24%, respectively, compared with the control.

**Table 6:** Tolerance of *L. salivarius* CGMCC20700 to pH and bile salts.

Treatment	Time (h)	Viable count (× 10^7^ CFU /mL)	Survival rate (%)
control	4	6.96 ± 0.31	100^1^
0.30% bile salt	4	4.63 ± 0.25	62.64^*^
0.60% bile salt	4	2.35 ± 0.12	33.76^**^
0.90% bile salt	4	1.81 ± 0.24	26.01^**^
pH 2	5	4.03 ± 0.36	57.91^*^
pH 3	5	5.84 ± 0.25	83.91^*^
pH 4	5	6.42 ± 0.38	92.24

1 Untreated control was considered 100%.

^*^p < 0.05; ^**^p < 0.01.

#### Auto aggregation and hydrophobic capability

According to the results, the aggregation rate of the *L. salivarius* CGMCC20700 strain was 57.12 ± 1.23%. The hydrophobicity of *the L. salivarius* CGMCC20700 strain was determined, and the hydrophobicity index was 61.16 ± 1.19%.

## Discussion

Probiotics are considered biotherapeutic agents owing to their potential to bestow various health benefits (Al-Khalaifa et al., 2019). Numerous studies have shown that probiotics may adhere and survive in the gastrointestinal tract of humans and animals and contribute to maintaining a microecological balance of the gut microbiome, promoting digestive and metabolic processes, and modulating the immune response, thereby enhancing host immunity and improving human and animal health (Kai et al., 2020; Zheng et al., 2021). However, since the effectiveness of probiotics is species or strain dependent, they should meet a series of specific characteristics, such as safety, functional and beneficial characteristics (Li et al., 2020; Wu et al., 2022). Thus, in this study, we focused on the safety and potential probiotic properties of *L. salivarius* CGMCC20700 using a series of *in vitro* tests combined with whole-genome sequencing to reveal their potential biological functions.

The development of new strain resources and evaluation of the safety of strains is necessary to obtain the most effective probiotics (Kai et al., 2020; Wu et al., 2022). It has been reported that candidate probiotics should not transport antibiotic resistance genes for hosts (García-Hernández et al., 2016; Goel et al., 2020). However, previous studies have found that LAB strains may develop resistance to tetracycline, 4-quinolones, rifampicin, and macrolides due to ribosome protection, antibiotic efflux and associated efflux pump formation (Ma et al., 2021; Fu et al., 2022). In this study, the ARDB and a variety of antibiotic susceptibility tests found that the strain contained macrolide and tetracycline antibiotic genes and was sensitive to tetracycline and erythromycin, showing antibiotic resistance similar to or lower than that of other known probiotic strains (Zheng et al., 2021; Fu et al., 2022). For instance, *E. lactis* JDM1 was resistant to erythromycin, quinupristin-dalfopristin 1R and furantoin and contained six highly similar resistance genes, efmA, aac and msrC (Fu et al., 2022); *Lactobacillus paracasei* CY2 is resistant to four types of antibiotics, kanamycin, gentamicin, and vancomycin (Zheng et al., 2021). Furthermore, the presence of virulence factors and hemolytic activity are important indicators of potentially beneficial strains (Goel et al., 2020; Kai et al., 2020). The ARDB database and hemolytic tests revealed that *L. salivarius* CGMCC20700 lacked highly similar virulence factor genes and showed nonhemolytic activity, which implied that the strains were not toxic. In particular, *Lactobacillus* virulence determinants that were confirmed by previous studies include cytohemolysin (cyl), aggregates (AS), and gelatinases (Kai et al., 2020; Vyacheslav et al., 2020); however, none of these were identified in *L. salivarius* CGMCC20700. Additionally, no harmful effects were found on the growth performance and overall health of broilers after feeding a *L. salivarius* CGMCC20700 supplementary diet. Thus, these results comprehensively indicated that *L. salivarius* CGMCC20700 has good safety for use in livestock and poultry farming.

Antimicrobial activity is one of the most important criteria for selecting new probiotic strains because probiotics can maintain intestinal homeostasis by inhibiting the growth of intestinal pathogenic bacteria (Grosu-Tudor et al., 2014). Due to the convenience of whole-genome sequencing and the diversity of genome mining tools, it is possible to predict a strain’s capability for producing antimicrobial compounds (Fu et al., 2022; Wu et al., 2022). In this study, AntiSMASH 5.0 and BAGEL 4.0 prediction results showed that two putative genes associated with antimicrobial compounds, T3PKS and enteroccin A, were identified in the *L. salivarius* CGMCC20700 genome. Previous studies have shown that LAB are known to have a well-developed secretion system that can produce a variety of metabolites, including antimicrobial peptides synthesized by ribosomes (RiPPs), nonribosomal synthetic peptides (NRPs) and polyketides (PKs) (Weber et al., 2015). This evidence showed that *L. salivarius* CGMCC20700 was capable of producing a variety of antibacterial compounds to help livestock and poultry against pathogenic infections. Additionally, the cell-free supernatant of *L. salivarius* CGMCC20700 showed high antibacterial activity against uncommon types of pathogens, and the maximum inhibition circle size was up to 26 mm, which further demonstrated the antibacterial compounds produced by *L. salivarius* CGMCC20700 with excellent broad-spectrum antibacterial activity. Thus, these results demonstrated that *L. salivarius* CGMCC20700 could effectively influence the balance of the intestinal flora and occupy a good competitive position.

Moreover, tolerance to bile salts and acidic conditions are two key characteristics when assessing beneficial traits, as the presence of bile salts and highly acidic conditions constitute the greatest barriers to the survival of *Lactobacillus* in the animal host gastrointestinal tract (Kai et al., 2020; Vyacheslav et al., 2020). Previous studies have shown that the *dltA*, *dltD* and *rfbB* genes mainly contribute to acid tolerance and the survival of bacteria in acidic environments; the *rfbB* gene encodes dTDP-glucose 4,6-dehydratase activity and plays an important role in the response of bacteria to low-pH conditions (Behera et al., 2018; Goel et al., 2020). In this study, *L. salivarius* CGMCC20700 genomic analysis showed that all the above bile salt and acid tolerance-related genes were obtained and that the similarity of these genes was over 90%, implying that *L. salivarius* CGMCC20700 had good bile salt and acid tolerance. Similarly, after treatment with 0.90% bile salts for 4 h and pH = 2 for 5 h, the survival rate of *L. salivarius* CGMCC20700 was maintained at 26.01 and 57.91%, respectively, demonstrating higher tolerance efficacy compared to the other partial probiotic LAB strains. For instance, *Lactobacillus plantarum* CY2 and *Lactobacillus paracasei* CY3 isolated from yak milk were only maintained at 20.10 and 12.38%, respectively, after treatment with 0.5% bile salts for 4 h, and *L. paracasei* CY3 was only maintained at 36.09% after treatment at pH 2 for 3 h (Zheng et al., 2021). Thus, these findings confirmed that *L. salivarius* CGMCC20700 can survive typical animal gastrointestinal tract conditions.

Additionally, self-agglomeration and hydrophobicity are also important characteristics for the efficient colonization of probiotics in the animal gut (Goel et al., 2020; Qureshi et al., 2020). In this study, the self-agglomeration and hydrophobicity of *L. salivarius* CGMCC20700 were 57.12 and 61.16%, respectively. Furthermore, an anti-pathogenic effect and adhesion ability with related genes were also identified in the *L. salivarius* CGMCC20700 genome, which associated functional genes involved *Lux S*, *Mucin22* and *fbp*. Generally, *Mucin22* and *fbp* genes are responsible for adhesion ability to the intestinal epithelial layer, likely excluding the adhesion of pathogenic species (Garcia-Gonzalez et al., 2018). Additionally, the genome of *L. salivarius* CGMCC20700 also contains *dltB* and *dltD* genes, and these genes are involved in human immunity and anti-inflammatory processes (Elbanna et al., 2018; Galdeano et al., 2019; Goel et al., 2020). Collectively, the combined *in vitro* probiotic characterization and genetic analysis showed that *L. salivarius* CGMCC20700 has good potential as a probiotic with resistance to intestinal and gastric fluids, adherence to intestinal epithelial tissues and robust immunomodulatory and anti-inflammatory effects. Notably, the probiotic potential of *L. salivarius* CGMCC20700 needs to be systematically investigated in further experiments, both at the cellular level and *in vivo* in animal experiments.

## Conclusion

In the present study, we identified a *L. salivarius* CGMCC20700 strain and investigated its safety and probiotic properties. The genome screenings indicated the absence of active antibiotic resistance genes and virulence factor genes. Hemolytic assays, acute oral toxicology, and antibiotic resistance tests further confirmed its safety. The detection of antimicrobial gene clusters, adhesion-related genes and stressor-reducing genes, such as extreme acids and bile salts, and the simulation of gastric and intestinal fluid stresses revealed potential probiotic properties. Additionally, *L. salivarius* CGMCC20700 is highly self-agglomerative and hydrophobic, and *in silico* analysis demonstrated the genes responsible for adhesion, immunity, and anti-inflammation. Collectively, this study provides experimental evidence that *L. salivarius* CGMCC20700 can serve as an effective probiotic candidate to replace antibiotic applications in livestock and poultry farming.

## Data availability statement

The datasets presented in this study can be found in online repositories. The names of the repository/repositories and accession number(s) can be found in the article/Supplementary material.

## Author contributions

Y-HJ: investigation, methodology, data curation, software, and writing review and editing. R-SY: methodology, investigation, and writing review and editing. Y-CL: methodology and writing review and editing. W-GX: methodology, investigation, data curation, and software. H-YZ, FW, and Q-LZ: methodology and investigation. L-BL: conceptualization, methodology, resource, data curation, writing original draft, and writing review and editing. All authors contributed to the article and approved the submitted version.

## Funding

This study was supported by Yunnan Major Scientific and Technological Projects (grant no. 202202AG050008).

## Conflict of interest

The authors declare that the research was conducted in the absence of any commercial or financial relationships that could be construed as a potential conflict of interest.

## Publisher’s note

All claims expressed in this article are solely those of the authors and do not necessarily represent those of their affiliated organizations, or those of the publisher, the editors and the reviewers. Any product that may be evaluated in this article, or claim that may be made by its manufacturer, is not guaranteed or endorsed by the publisher.

## Footnotes

^1^http://topaz.gatech.edu/GeneMark/

^2^https://antismash.secondarymetabolites.org

^3^http://bagel4.molgenrug.nl/index.php
