# Supplementary material for: Assessment of the safety and probiotic characteristics of Lactobacillus salivarius CGMCC20700 based on whole-genome sequencing and phenotypic analysis
Source: Front Microbiol. 2023 Mar 16;14:1120263. doi: 10.3389/fmicb.2023.1120263 (PMC10062426; doi:10.3389/fmicb.2023.1120263)
Supplement: Supplementary file 1 [file Table_1.DOCX]

**Table S1 Putative antibiotic resistance genes identified in the genome of *L. salivarius* CGMCC20700.**

| **Gene ID** | **Gene** | **Antibiotics** | **Identity(%)** |
| --- | --- | --- | --- |
| L.S.GL000022 | mdtg | deoxycholate,fosfomycin | 42.8 |
| L.S.GL000041 | vanrd | vancomycin,teicoplanin | 41.4 |
| L.S.GL000077 | lsa | lincosamide,streptogramin_b,macrolide | 50.5 |
| L.S.GL000221 | vanrg | vancomycin | 45.7 |
| L.S.GL001060 | vanre | vancomycin | 41.6 |
| L.S.GL001351 | mdtg | deoxycholate,fosfomycin | 48.6 |
| L.S.GL001735 | ermc | lincosamide,streptogramin_b,macrolide | 99.6 |
| L.S.GL001751 | bcra | bacitracin | 52.5 |
| L.S.GL001755 | tetm | tetracycline | 95.6 |
| L.S.GL001756 | tetl | tetracycline | 98.5 |

**Table S2 Putative virulence factors in the *L. salivarius* CGMCC20700 genome.**

| **Gene ID** | **Gene** | **Predicted functions** | **Identity(%)** |
| --- | --- | --- | --- |
| L.S.GL000325 | clpE | ATP-dependent protease | 60.8 |
| L.S.GL000456 | lisR | two-component response regulator | 79.4 |
| L.S.GL000497 | cpsA | undecaprenyl diphosphate synthase | 64.8 |
| L.S.GL000575 | tig/ropA | Trigger factor, putative | 60.0 |
| L.S.GL000577 | gnd | 6-phosphogluconate dehydrogenase | 68.7 |
| L.S.GL001041 | eno | Enolase, putative | 72.0 |
| L.S.GL001044 | plr/gapA | glyceraldehyde-3-phosphate dehydrogenase | 60.1 |
| L.S.GL001046 | clpP | ATP-dependent Clp protease proteolytic subunit | 72.6 |
| L.S.GL001055 | lgt | lgt | 61.5 |
| L.S.GL001072 | groEL | chaperonin GroEL | 69.9 |
| L.S.GL001130 | SMU.322c | glucose-1-phosphate uridylyltransferase | 75.3 |
| L.S.GL001386 | cpsI | UDP-galactopyranose mutase | 64.1 |
| L.S.GL001404 | SMU.824 | dTDP-4-keto-L-rhamnose reductase | 60.9 |
| L.S.GL001405 | STER_1222 | dTDP-D-glucose 4,6-dehydratase | 79.4 |

Note: only listed the annotated genes with more than 60% similarity.
